# Associations between indicators of socioeconomic position and DNA methylation: a scoping review

**DOI:** 10.1186/s13148-021-01189-0

**Published:** 2021-12-14

**Authors:** Janine Cerutti, Alexandre A. Lussier, Yiwen Zhu, Jiaxuan Liu, Erin C. Dunn

**Affiliations:** 1 Department of Pscyhology, University of Vermont, 2 Colchester Ave, Burlington, VT USA; 2 Psychiatric and Neurodevelopmental Genetics Unit, Center for Genomic Medicine, Massachusetts General Hospital, 185 Cambridge Street, Simches Research Building 6th Floor, Boston, MA 02114 USA; 3 Department of Epidemiology, Harvard T.H. Chan School of Public Health, Boston, MA USA; 4 Department of Psychiatry, Harvard Medical School, Boston, MA USA; 5 Stanley Center for Psychiatric Research, The Broad Institute of Harvard and MIT, Cambridge, MA USA

**Keywords:** Socioeconomic factors, DNA methylation, Epigenetics, Systematic review

## Abstract

**Background:**

Socioeconomic position (SEP) is a major determinant of health across the life course. Yet, little is known about the biological mechanisms explaining this relationship. One possibility widely pursued in the scientific literature is that SEP becomes biologically embedded through epigenetic processes such as DNA methylation (DNAm), wherein the socioeconomic environment causes no alteration in the DNA sequence but modifies gene activity in ways that shape health.

**Methods:**

To understand the evidence supporting a potential SEP-DNAm link, we performed a scoping review of published empirical findings on the association between SEP assessed from prenatal development to adulthood and DNAm measured across the life course, with an emphasis on exploring how the developmental timing, duration, and type of SEP exposure influenced DNAm.

**Results:**

Across the 37 identified studies, we found that: (1) SEP-related DNAm signatures varied across the timing, duration, and type of SEP indicator; (2) however, longitudinal studies examining repeated SEP and DNAm measures are generally lacking; and (3) prior studies are conceptually and methodologically diverse, limiting the interpretability of findings across studies with respect to these three SEP features.

**Conclusions:**

Given the complex relationship between SEP and DNAm across the lifespan, these findings underscore the importance of analyzing SEP features, including timing, duration, and type. To guide future research, we highlight additional research gaps and propose four recommendations to further unravel the relationship between SEP and DNAm.

**Supplementary Information:**

The online version contains supplementary material available at 10.1186/s13148-021-01189-0.

## Introduction

Socioeconomic position (SEP) is commonly measured across health-related fields, as it is considered “a fundamental cause of disease” [1, 2]. SEP is a multidimensional concept, encompassing diverse social and economic components, such as actual resources (e.g., weekly income) and rank-based characteristics (e.g., occupational prestige) [3, 4]. These components can be measured at the individual or aggregate level (e.g., household, neighborhood) and are often quantified by indicators such as education, income, and housing conditions [1, 5, 6].

Decades of observational studies have shown that low SEP is strongly associated with adverse behavioral and health outcomes among children and adults, including the 14 major cause-of-death categories worldwide [2, 7–13]. Evidence from experimental and quasi-experimental studies also suggests that SEP may play a *causal* role in these outcomes [14, 15]. Indeed, interventions and policies that provide food [16], housing [17], medical-care subsidies [18], or income-transfer supplements [19] have demonstrated widespread positive effects on health, emotional, behavioral, educational, and employment outcomes, while also reducing risk for psychiatric disorders, substance use, and criminal behavior. As one example, a natural experiment of children whose families received annual income supplements showed a 40% decrease in child psychopathology compared to the 4 years before receiving supplements [20], with the protective effect of financial assistance persisting into early adulthood [21].

Although prior studies have established SEP as a potent determinant of health, the biological mechanisms explaining this relationship are not well understood. One widely pursued hypothesis is that SEP may alter gene expression and subsequent long-term health through changes in DNA methylation (DNAm) levels, an epigenetic mechanism wherein methyl groups are added to or removed from cytosine residues in DNA, typically in cytosine–guanine (CpG) dinucleotides [22, 23].

Three common approaches for measuring DNAm levels include: global, candidate gene, and epigenome-wide methods. Global DNAm studies measure overall DNAm levels via a wide array of commonly used techniques, including high-performance liquid chromatography, polymerase chain reaction (PCR), enzyme-linked immunosorbent assay (ELISA), or mass spectrometry-based methods. Although many global methods are relatively fast and easy to use, their measures are often imprecise, providing only a rough estimation of global DNAm content [24]. Candidate gene studies measure DNAm variation at a set of loci located in specific genes/regions of interest via techniques like bisulfite sequencing and array or bead hybridization [24]. Candidate studies are typically inexpensive and straightforward to perform; however, given the small number of loci tested and the large amount of DNAm variation along the epigenome, they often suffer from a number of limitations related to reliability and systematic bias, making their findings difficult to interpret and reproduce across studies [25]. Epigenome-wide studies (EWASs) measure DNAm variations at large-scale coverage of hundreds of thousands of loci across the epigenome via high-throughput array- and sequencing-based technologies [26]. Although this approach is useful for exploratory analyses and comprehensive studies, EWASs are expensive to conduct and, given the number of loci tested, require large sample sizes to detect associations with relatively small effect sizes.

To better understand the role of SEP on epigenetic processes, we performed a scoping review of empirical studies (global, candidate gene, and EWAS) investigating the association between SEP and DNAm in humans. Prior reviews on this topic have focused on a narrow subset of SEP indicators (e.g., neighborhood disadvantage) [27], mechanisms to investigate epigenetic changes (e.g., epigenetic clock, telomere attrition) [28], or specific time periods in the life course (e.g., childhood) [29, 30]. Our aim, therefore, was to characterize the overall evidence on the association between SEP and DNAm, including diverse SEP indicators and DNAm approaches across the entire life course. We performed a scoping review, rather than a systematic review or meta-analysis, because our goal was to provide a comprehensive overview of the evidence on a research topic and address broader research questions related to that topic, instead of answering a specific question through systematic qualitative or quantitative assessments [31].

Our scoping review was organized by four main overarching research questions: (1) What are the characteristics of published studies on the relationship between SEP and DNAm; (2) What is the overall state of the evidence on the SEP-DNAm relationship; (3) Does the timing and/or duration of SEP influence DNAm patterns; and (4) Do different SEP indicators show differential DNAm profiles?

Although prior studies have shown that low SEP is especially harmful when experienced early in development and chronically throughout childhood [12, 21], we are unaware of any attempts to identify and compare findings between studies to determine whether there are trends in the literature suggesting specific ages or sensitive periods during development when SEP-induced DNAm changes are most likely to occur. Furthermore, studies analyzing multiple SEP indicators have found that individual SEP exposures may play related yet distinct roles in health and behavioral outcomes [32–35]. However, no prior reviews have examined whether there is converging evidence across studies that different SEP indicators associate with distinct patterns of DNAm changes. Answers to these research questions will not only provide a better understanding of how aspects of the socioeconomic environment become biologically embedded across the lifespan, but will also help to guide future research to facilitate targeted interventions aimed at reducing the negative sequelae of low SEP.

## Methods

We performed this review in accordance with the preferred reporting items for systematic reviews and meta-analyses extension for scoping reviews (PRISMA-ScR) guidelines [36]. Due to the substantial heterogeneity of study characteristics, designs, and methods, we conducted a narrative synthesis, rather than a meta-analysis, to summarize findings across studies [36–38]. We did not assess risk of publication bias, because most standard systematic review indices evaluating risk of bias were not applicable to the observational studies included here [39, 40]. Furthermore, our objective was not to critically appraise individual studies to determine robustness or minimize biases for any subsequent data synthesis. Instead, we sought to map the evidence across studies, providing a narrative synthesis with an eye toward identifying key features and trends.

### Study identification

We systematically identified articles published from inception through September 18, 2019 (date last searched), on PubMed and PsycINFO. We worked closely with an experienced reference librarian to develop a combination of database-specific index terms (e.g., ‘socioeconomic factors,’ ‘epigenomics’) and individual terms located in the title or abstract (e.g., ‘income,’ ‘occupation,’ ‘epigenetics’), which were further refined through team discussion (see Additional file 1 for final search terms). We also assessed reference lists of review articles and included additional relevant studies.

### Study selection

We included only human empirical studies that examined an independent association between SEP and DNAm, including global, candidate gene, and epigenome-wide approaches (see Additional file 1 for inclusion and exclusion criteria). An independent reviewer evaluated the titles and abstracts of all publications identified by our search and then reviewed the full texts of relevant publications to determine eligibility. We resolved any uncertainty on study eligibility by discussion with three other team members.

### Data extraction process

An independent reviewer extracted the data (in triplicate), discussed the results with team members, and continually updated the data in an iterative process based on team discussions. Three other independent reviewers verified data extraction results; any disagreements were resolved by consensus and team discussions. We extracted the following information from each study: (1) sample features (i.e., sample size, cohort name, sex, race/ethnicity, country of enrollment); (2) overarching research question and design; (3) SEP exposure features; (4) approach to analyzing DNAm (DNAm approach; i.e., global, candidate gene, epigenome-wide association); (5) DNAm assessment age(s); (6) tissue type(s) investigated; (7) DNAm measurement method; (8) covariates; (9) SEP-DNAm associations examined; and (10) primary and secondary findings. Of note, we defined “global” DNAm as measures or estimates of the overall DNA methylome, including DNA methylation levels of repetitive elements (e.g., LINE-1 and Alu) [41].

To synthesize how studies conceptualized SEP and to compare between different overarching SEP aspects, we categorized each SEP measure (referred to throughout this review as “indicator”) into one of the following domains: education, occupation, income, neighborhood, subsidy, composite (i.e., aggregated SEP measure), and other. Additionally, we reported how each SEP indicator was captured, specifically the method of data collection (e.g., subjective self-report versus objective census-tract data; retrospective versus prospective) and also the measurement scale (e.g., dichotomous, categorical, continuous) used to classify individual low to high SEP status. Detailed information on SEP features is included in Additional file 1.

To compare results more consistently across studies, we extracted results of SEP-DNAm associations reported from the most stringent significance test within the simplest (or unadjusted) regression model. We recorded the direction of association with DNAm (lower SEP associated with increase/decrease in DNAm), if reported, in our main results. For the nine epigenome-wide association studies (EWAS) that used the Illumina Human Methylation 450k array (450k array) method, we compiled all individual CpG site (CpGs) IDs analyzed and corresponding *p* value and adjusted *p* value (after multiple testing procedure). We contacted authors of seven studies for these summary statistics and retrieved the summary statistics online for the other two papers. No other contact with authors was made.

### Data analysis

To address our first research question (what are the overall characteristics of studies on SEP and DNAm?), we conducted descriptive analyses to summarize characteristics of all studies included and reported average sample size, distribution of overall study characteristics, and individual-level study methods and results grouped by DNAm approach. We addressed our second research question (what is the overall state of the evidence on the relationship between SEP and DNAm?) by describing the overall state of study findings, grouped by their approach to measuring DNAm. To address our third question (does the timing and/or duration of SEP influence DNAm profiles?), we performed a qualitative analysis to compare results across studies analyzing SEP at different exposure ages (i.e., childhood, adulthood, or both) and DNAm. A meta-analysis was not feasible given the heterogeneity in SEP measures and effect estimates reported, along with differences in underlying samples and study designs. Instead, we provided a narrative synthesis of these study findings, summarizing the SEP-DNAm associations found in each age group within and across studies. We addressed our fourth question (do different SEP indicators show differential DNAm profiles?) using summary statistics from EWAS 450k array studies described above (Additional file 1).

## Results

**Fig. 1:**
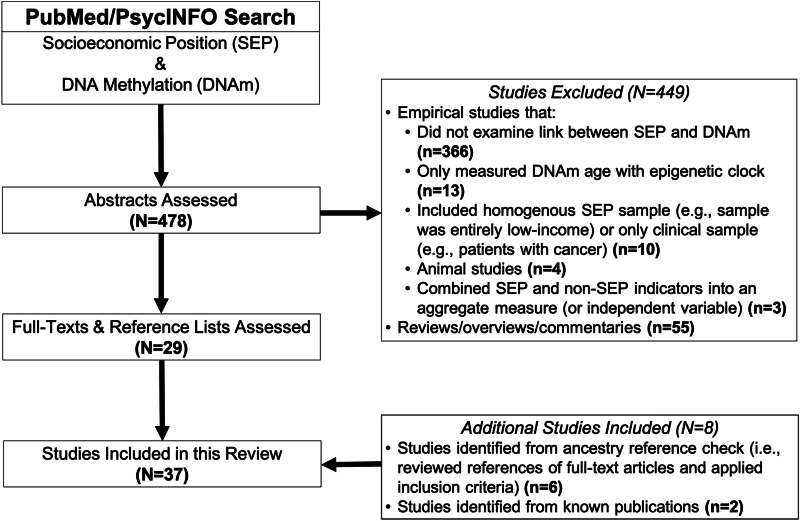
Systematic search and selection procedure. The full search and selection procedure of published studies from inception through September 2019 for a systematic review of the association between socioeconomic position (SEP) and DNA methylation (DNAm). A PubMed and PsycINFO search returned 478 articles. Abstracts were assessed and most articles (*n* = 366) were excluded because they did not include an SEP measure as an exposure and/or did not include DNAm as an outcome. Thirteen articles only measured “epigenetic age,” or estimates of biological age based on DNAm, and were also removed. Another 10 were removed because they did not include a healthy control group or their sample was homogenous for SEP level (e.g., entirely low income). Four animal studies were removed. Three studies were excluded because they combined SEP and non-SEP (e.g., childhood abuse) measures into one aggregated composite measure. Fifty-five were reviews, overviews, or commentaries and were also excluded. We identified six additional studies by reviewing reference lists of 29 eligible publications and added two known publications to the review. In final, 37 studies were included in this systematic review. *Note*: Excluded studies do not sum to 449 due to overlap

Our search returned 478 results; see Fig. 1 for flowchart of entire search and selection procedure. A total of 37 studies met our inclusion criteria, capturing global DNAm (*n* = 7; Table 1) [42–48], candidate gene (*n* = 18; Table 2) [49–66], and EWAS (*n* = 12; Table 3) studies [67–78]. Detailed information on each study is provided in Additional file 2: Tables S1–S3. Since 2008 (the date of the first published paper included in our review), there has been a steady growth in the number of studies investigating the SEP-DNAm relationship. Specifically, EWAS and candidate gene studies rose, while global DNAm studies steadily plateaued (Additional file 1: Fig. S1).

**Table 1:** Associations between socioeconomic position and DNA methylation from global DNA methylation studies (*n* = 7)

References	*N*	SEP indicator		DNAm		Effect^a^			
		Exposure age(s)^b^	SEP domain(s)^c^	Assessment age(s)^b^	DNAm measure	↑↓		Associated SEP domain	Significance threshold
Coker et al. [42]	241	Prenatal	Ed., In., Neigh	Birth	% DNAm of LINE-1 and Alu elements	↑	LINE-1	Neigh	*p* = 0.004
Herbstman et al. [43]	279	Prenatal	Ed., Misc., Sub	Birth	% Global DNAm	No findings at *p* ≤ 0.10			
	165			Child					
Perng et al. [44]	568	Child	Comp., Ed	Child	% DNAm of LINE-1 elements	↓	LINE-1	Ed	*p* trend = 0.06
Terry et al. [45]	92	Child	Comp	Adult	Disintegrations per minute per microgram DNA	No findings at *p* < 0.05			
Subramanyam et al. [46]	988	Life course	Ed., In., Misc	Adult	% DNAm of LINE-1 and Alu elements	↑	LINE-1	Misc. (Adult)	*p* < 0.01
						↓	Alu	Misc. (Adult)	*p* < 0.01
Tehranifar et al. [47]	90	Life course	Ed., In., Occ., Misc	Adult	% DNAm of LINE-1, Alu, and Sat-2 elements	↑	Sat2	Ed. (Child)	*p* < 0.05
						↑	Alu	Misc. (Child)	*p* < 0.05
						↑	LINE-1	In. (Adult)	*p* < 0.05
						Nonlinear with Alu		In. (Adult)	*p* < 0.05
McGuinness et al. [48]	239	Adult	Ed., In., Neigh., Misc	Adult	% Global DNAm	↓		Ed	*p* < 0.05
						↓		Occ	*p* < 0.05
						↓		Neigh	*p* < 0.05

Studies presented in this table are shown in order of DNAm assessment age, then by SEP exposure age followed by alphabetically. For individual-level study details, including covariates, see Additional file 2: Table S1

*Assess.* assessments, *DNAm* DNA methylation, *Ed.* education, *In.* income, *Occ.* occupation, *Misc.* miscellaneous (i.e., “other” domain), *Neigh.* neighborhood, *Sub.* subsidy, *SEP* socioeconomic position

^a^Effects reported within the simplest (or unadjusted) model. General direction of effect for association between DNAm and SEP measure reported by arrows, indicating increased or decreased DNAm levels for low SEP. Associated SEP domain reported with exposure age provided in parenthesis if both child and adult SEP exposures were analyzed. *p* Values reported for significance threshold

^b^SEP exposure and DNAm assessment ages are reported by life course group: prenatal (< 0 years), birth (~ 0 years), child (0–18 years), adult (18+ years). “Life course” indicates ages of exposure spanned prenatal, birth, or childhood to adulthood

^c^The type of SEP domains covered by SEP indicators included in each study to assess socioeconomic factors. For full list of SEP indicators and domains included by individual studies, see Additional file 2: Table S1

**Table 2:** Associations between socioeconomic position and DNA methylation from candidate gene studies (*n* = 18)

References	*N*	SEP indicator		DNAm		Effect^a^			
		Exposure age(s)^b^	SEP domain(s)^c^	Assessment age(s)^b^	Targeted gene(s)	↑↓	Associated gene	Associated SEP domain	Significance threshold
King et al. [49]	489	Prenatal	Ed., In	Birth	9 genes	↓	IGF2	Ed./In	*p* < 0.05
						↓	H19	Ed	*p* < 0.05
						↓	MEG3	Ed./In	*p* < 0.05
King et al. [50]	489	Prenatal	Neigh	Birth	MEG3	↑	MEG3	Neigh	*p* = 0.002
Appleton et al. [51]	444	Prenatal, Birth	Comp., Ed., Misc	Birth	HSD11B2	↓	HSD11B2	Comp	*p* < 0.05
						↓	HSD11B2	Ed	*p* < 0.05
						↓	HSD11B2	Misc	*p* < 0.05
Piyasena et al. [52]	50	Birth	Neigh	Birth, Child 2×	IGF2; H19; FKBP5	↓	IGF2	Comp	*p* < 0.05
						↓	FKBP5	Comp	*p* < 0.05
Obermann-Borst et al. [53]	120	Child	Ed	Child	IGF2; IGF2R; INSIGF	↑	INSIGF	Ed	*p* = 0.021
Obermann-Borst et al. [54]	120	Child	Ed	Child	LEP	↑	LEP	Ed	*p* = 0.008
Wrigglesworth et al. [55]	33	Child	Neigh	Child	BDNF IV	↑	BDNF IV	Neigh	*p* = 0.0001
Huang et al. [56]	613	Birth	Ed., Occ	Adult	5 genes	↓	ABCA1	Occ	*p* = 0.03
						↓	HSD11B2	Ed	*p* = 0.01
McDade et al. [57]	494	Child 4×	Misc	Adult	114 genes	↑	GNG2	Misc	*q* = 0.0093
						↓	C1S	Misc	*q* = 0.0093
Loucks et al. [58]^d^	141	Child	Comp	Adult	198,224 CpGs	↑↓	162 CpGs	Comp	*p* < 0.001
Needham et al. [59]	1264	Life course	Ed	Adult	18 genes	↑	7 genes	Ed. (Child)	*q* < 0.20
						↑	6 genes	Ed. (Adult)	*q* < 0.20
						↑	10 genes	Ed	*q* < 0.20
Smith et al. [60]	1226	Adult	Neigh	Adult	18 genes	↑↓	12 genes	Neigh	*q* ≤ 0.10
Stringhini et al. [61]	857	Life course	Occ	Adult	17 genes	↑↓	2 genes	Occ. (Adult)	*q* ≤ 5.10 × 10^−3^
						↓	6 genes	Occ	*q* ≤ 1.49 × 10^−3^
Jones-Mason et al. [62]^e^	100	Adult	Comp	Adult	SLC6A4	↑	SLC6A4	Comp	*q* < *0.05*
Kogan et al. [63]	309	Adult	Comp	Adult	OXTR	↑	OXTR	Comp	*p* < 0.01
de Rooij et al. [64]	675	Adult	Comp., Ed	Adult	GR 1-C	↓	GR 1-C	Ed	*p* = 0.03
Simons et al. [65]	100	Adult 3×	Comp	Adult	OXTR	↑	OXTR	Comp	*p* ≤ 0.01
Swift-Scanlan et al. [66]	48	Adult	Comp	Adult	COMT	No findings at *q* < 0.05			

Studies presented in this table are shown in order of DNAm assessment age, then by SEP exposure age followed by alphabetically. For individual-level study details, including covariates and number of CpG sites targeted within each gene, see Additional file 2: Table S2

*Comp.* composite, *CpGs* CpG sites, *DNAm* DNA methylation, *Ed.* education, *In.* income, *Occ.* occupation, *Misc.* miscellaneous (i.e., “other” domain), *Neigh.* neighborhood, *SEP* socioeconomic position

^a^Effects reported from the most stringent significance test within the simplest (or unadjusted) model. General direction of effect for association between DNAm and SEP measure reported by arrows, indicating increased or decreased DNAm levels for low SEP. Associated SEP domain reported with exposure age provided in parenthesis if both child and adult SEP exposures were analyzed. *p *Values reported for significance threshold; *q*-values indicate *p* values corrected for multiple testing using the false discovery rate (FDR) method

^b^SEP exposure and DNAm assessment ages are reported by life course group: prenatal (< 0 years), birth (~ 0 years), child (0–18 years), adult (18+ years). Number of assessments indicated (e.g., 2×, 3×) if SEP or DNAm was measured at more than one timepoint per life course group. “Life course” indicates ages of exposure spanned prenatal, birth, or childhood to adulthood

^c^The type of SEP domain covered by SEP indicators included in each study to assess socioeconomic factors. For full list of SEP indicators and domains included by individual studies, see Additional file 2: Table S2

^d^Loucks et al. [58] were included in candidate gene section because study assessed SEP-DNAm associations only in CpG sites that were previously associated with BMI (FDR < 0.25) in an EWAS using the same sample

^e^Reported effect was found when sample was stratified by attachment styles (see Jones-Mason et al. [62] for more details)

**Table 3:** Associations between socioeconomic position and DNA methylation from epigenome-wide association studies (*n* = 12)

References	*N*	SEP indicator		DNAm		Effect^a^			
		Exposure age(s)^b^	SEP domain(s)^c^	Assessment age(s)^b^	Targeted CpGs	↑↓	Associated CpGs	Associated SEP indicator	Significance threshold
Santos et al. [76]	426	~ Birth	Comp., Ed., Misc., Sub	Birth	856,832	↑	1	Ed	*q* < 0.05
						↑↓	3	Misc	*q* < 0.05
						↑↓	6	Comp	*q* < 0.05
						↑↓	10	Misc	*q* < 0.05
Laubach et al. [73]	609	Prenatal	Comp., Ed., In., Misc., Neigh., Sub	Birth, Child 2×	Birth: 394,460	↑↓	3	Comp	*p*_bonf_ < 0.05
					3 yr: 394,460	↑	1	Comp	*p*_bonf_ < 0.05
					7 yr: 394,460		0		*p*_bonf_ < 0.05
Alfano et al. [67]	860	Prenatal	Ed., Occ	Birth, Child 2×	Birth: 285,021	↑↓	4	Ed	*q* < 0.05
					7 yr: 285,994		0		*q* < 0.05
					15 yr: 285,721	↑↓	20	Ed	*q* < 0.05
Bush et al. [70]	178	Child	Ed., In	Child	409,878	↑↓	1	Ed	*q* ≤ 0.05, delta ≥ 0.10
						↑↓	8	In	*q* ≤ 0.05, delta ≥ 0.10
Dunn et al. [71]	650	Child 5×	Neigh., Misc	Child	473,929	↑↓	10	Neigh	*p*_bonf_ < 1 × 10^−7^
						↑↓	9	Misc	*p*_bonf_ < 1 × 10^−7^
Beach et al. [68]	398	Child 3×	Comp	Adult	47,311	↑↓	2032	Comp	*q* < 0.05
Borghol et al. [69]	40	Life course	Comp	Adult	223,359	↑↓	1252	Comp. (Child)	*q* ≤ 0.20
						↑↓	545	Comp. (Adult)	*q* ≤ 0.20
Lam et al. [72]	92	Life course	Occ	Adult	22,922		0		*q* < 0.05
McDade et al. [75]	489	Life course	Ed., In., Misc	Adult	110,631	↑↓	2546	Comp	*q* < 0.05
						↑↓	1437	Ed	*q* < 0.05
						↑↓	817	Misc	*q* < 0.05
						↑↓	107	In	*q* < 0.05
Suderman et al. [77]	28	Life course	Comp., Ed., Misc	Adult	361,419	↑	2	Comp. (Child)	*q* < 0.20
						↑↓	3	Comp. (Adult)	*q* < 0.20
Karlsson Linnér et al. [74]^d^	10,767	Adult	Ed	Adult	442,227	↑↓	37	Ed	*p*_bonf_ < 1 × 10^−7^
van Dongen et al. [78]^d^	4152	Adult	Ed	Adult	410,746	↑	58	Ed	*p*_bonf_ < 1.2 × 10^−7^

Studies presented in this table are shown in order of DNAm assessment age, then by SEP exposure age followed by alphabetically. For individual-level study details, see Additional file 2: Table S3

*Comp.* composite, *CpGs* CpG sites, *DNAm* DNA methylation, *Ed.* Education, *In* income, *Misc.* miscellaneous (i.e., “other” domain), *Neigh.* neighborhood, *Sub.* subsidy, *SEP* socioeconomic position, *Yr* years

^a^Effects reported from the most stringent significance test within the simplest (or unadjusted) model. General direction of effect for association between DNAm and SEP measure reported by arrows, indicating increased or decreased DNAm levels for low SEP. Associated SEP domain reported with exposure age provided in parenthesis if both child and adult SEP exposures were analyzed. Here, *q*-values and *p*_bonf_-values indicate significance threshold after *p* values were corrected for multiple testing using the false discovery rate (FDR) and Bonferroni methods, respectively; delta value indicates delta beta (DNAm difference between the minimum and maximum levels of SEP measure) threshold

^b^SEP exposure and DNAm assessment ages are reported by life course group: prenatal (< 0 years), birth (~ 0 years), child (0–18 years), adult (18+ years). Number of assessments indicated (e.g., 2×, 3×) if SEP or DNAm was measured at more than one timepoint per life course group. “Life course” indicates ages of exposure spanned prenatal, birth, or childhood to adulthood

^c^The type of SEP domain covered by SEP indicators included in each study to assess socioeconomic factors. For full list of SEP indicators and domains included by individual studies, see Additional file 2: Table S3

^d^Meta-analysis

### (1) What are the overall characteristics of studies on SEP and DNAm?

#### Sample features

Nearly all studies (92%; *n* = 34) analyzed samples from observational cohort studies, which collected data from participants either retrospectively or prospectively over a period of time. Specifically, 39 distinct cohorts were sampled in the current review, of which 16 were birth cohorts. Sample sizes varied widely across studies, ranging from 28 to 1264 participants (mean = 400). Studies were generally balanced with respect to sex (55% female on average), although eight studies included primarily (> 70%) or entirely female samples and four studies included primarily or entirely male samples. Over half of the studies (54%; *n* = 20) sampled participants solely from the USA, while others covered populations from Europe, Canada, Australia, the Philippines, Colombia, and Israel. Most studies focused on multi-ethnic samples (27%; *n* = 10) or White/majority White samples (24%, *n* = 9), while others included exclusively non-White samples (19%; *n* = 7) or Jewish ethnicity (3%; *n* = 1). The remaining 10 studies (27%) did not directly report race/ethnicity for their sample. Few studies (32%; *n* = 12) captured a wide range of SEP that included participants from very low/low to high SEP (Additional file 1).

#### Overarching research design

**Fig. 2:**
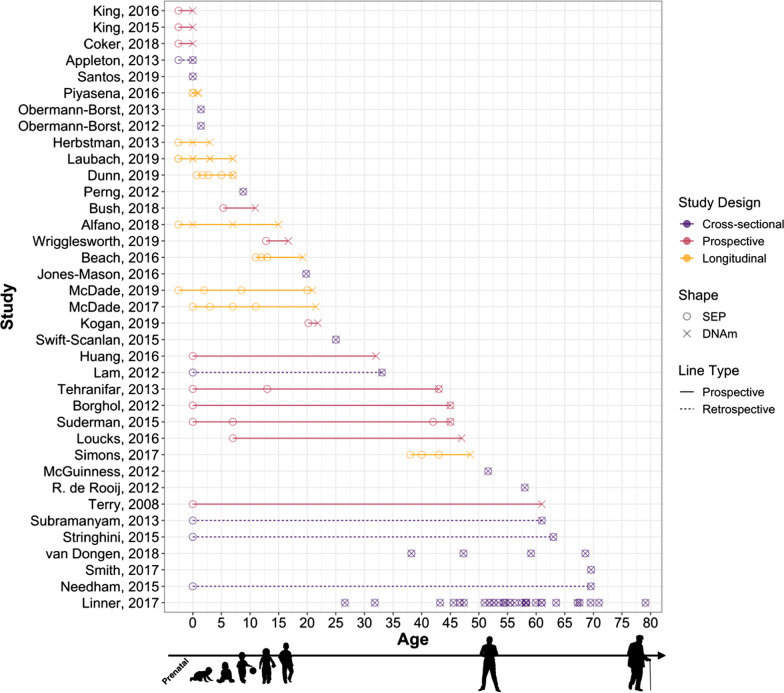
The stages in the life course captured by socioeconomic position exposure and DNA methylation assessment. The stages in the life course captured by socioeconomic position (SEP) exposure age and age at DNA methylation (DNAm) assessment are plotted by study design (cross-sectional, prospective, longitudinal) across all 37 studies included in review. Life-course stages include prenatal (< 0), birth (0), child (0–18 years), and adult (18+ years). Cross-sectional studies captured SEP exposure(s), and assessed DNA methylation at the same time in the life course; prospective studies prospectively assessed SEP exposure(s) no more than once over the life course; longitudinal studies prospectively assessed the same SEP exposure(s) at least twice over the life course. Solid lines indicate SEP was prospectively assessed, while dotted lines indicate SEP was retrospectively captured. *Note*: Karlsson Linnér et al. and Van Dongen et al. were meta-analyses

Most studies focused on associations between SEP and DNAm at a single time point, analyzed either cross-sectionally (43%; *n* = 16) or prospectively (32%; *n* = 12); the remaining 24% (*n* = 9) were longitudinal, assessing the same SEP exposure(s) repeatedly across time and/or repeated DNAm measures (Fig. 2). Of note, two cross-sectional meta-analyses analyzed cohort-level summary statistics on the association between adult educational attainment and DNAm. Slightly more than half of the studies (54%; *n* = 20) included SEP exposure measured either prenatally, at birth, or during childhood, with another nine (24%) focusing on SEP in adulthood. The remaining eight (22%) studies captured SEP exposure across the life course (i.e., spanning prenatal, birth, or childhood to adulthood), although four of these studies measured childhood SEP retrospectively in adulthood.

#### SEP exposure features

Across all 37 studies, a total of 96 SEP indicators were individually analyzed, tapping seven different domains: education (*n* = 28 indicators), composite (*n* = 17), occupation (*n* = 12), other (e.g., crowded dwelling, household assets, poverty status; *n* = 13), neighborhood (*n* = 12), income (*n* = 10), and subsidy (i.e., eligible for a form of public assistance; *n* = 4) (Additional file 1: Fig. S2). The number of SEP indicators analyzed across studies ranged from 1 to 7 (median = 2). Of the 17 studies analyzing composite measures, six additionally analyzed each indicator within the composite. Moreover, seven studies analyzed separate SEP indicators operating at more than one socioecological level (e.g., household, neighborhood) within the same assessment or time period.

Education-related measures were most commonly assessed across all three DNAm approaches (Additional file 1: Fig. S2A). Childhood, including birth, was the most common life-course period examined for SEP exposure (34% of indicators), most often through indices of parent- or household-level education, followed by the prenatal period (32%) and adulthood (27%; Additional file 1: Fig. S2B). Only 6% of indicators captured life-course SEP, spanning early life to adulthood. Most indicators were collected subjectively through caregiver (52%), self- (30%), or multigenerational reports (< 1%); 11% of indicators were assessed objectively via census-tract data (Additional file 1: Fig. S2C). Lastly, SEP indicators varied with respect to the measurement scale used to classify individual low to high SEP status for analysis, yet dichotomous measures (47%) were most common (Additional file 1: Fig. S2D).

#### DNAm approach

Candidate gene studies (49%; *n* = 18) were the most common study design, followed by EWAS (32%; *n* = 12) and global DNAm studies (19%; *n* = 7). Most studies assessed DNAm at a single time point in the life course: adulthood (57%; *n* = 21), childhood (19%; *n* = 7), or birth (14%; *n* = 5). Four studies (11%) assessed DNAm both at birth and during childhood. Whole blood was the most commonly studied tissue type, used in nearly 30% of studies (*n* = 11) (Additional file 1: Fig. S3). Although most studies targeted one tissue type, five studies (14%) sampled two different tissues to compare between DNAm levels in their analyses.

### (2) What is the overall state of the evidence on the relationship between SEP and DNAm?

#### Global DNAm studies (*n* = 7)

Within the global group, studies used six different methods for DNAm analysis (Additional file 2: Tables S1). Only five studies reported an association with SEP: the four studies that measured repetitive elements (i.e., a method for estimating global DNAm) and one of the studies measuring DNAm by other global methods (Table 1). Significance thresholds and direction of effects were inconsistent between studies.

#### Candidate gene association studies (*n* = 18)

Within the candidate group, studies targeted a variety of gene regions in 274 unique candidate genes using four different methods for DNAm analysis; the majority measured DNAm with the MassARRAY EpiTyper (*n* = 8) or the 450k array (*n* = 6; Additional file 2: Tables S2). Candidate genes spanned various domains of functional and biological significance, including body mass index (BMI), stress and inflammation, appetite regulation, fat metabolism, and cardiometabolic processes. All but one candidate gene study reported an association between SEP and DNAm at one or more genes, although significance thresholds and direction of effects were inconsistent across studies (Table 2). Two stress-related genes, *OXTR* and *FKBP5,* were the genes most often studied (targeted by three studies each), with all studies reporting increased DNAm for *OXTR,* while the direction of DNAm differences for *FKBP5* was mixed across studies.

#### Epigenome-wide association studies (EWAS; *n* = 12)

The majority of EWAS used the 450k array (*n* = 9; Additional file 2: Tables S3). Studies in this category reported 23 different analyses of SEP and DNAm, with 8912 total associations passing their most stringent significance thresholds (Table 3). Of note, two studies only reported the total number of associations, making it impossible to assess unique CpG-level associations in the present review. The general direction of DNAm for lower SEP values also varied between studies, with 2685 CpGs showing increased methylation and 1825 showing decreased methylation; the direction of associations at the remaining 4402 CpGs was not reported at the individual CpG level.

### (3) Does the timing and/or duration of SEP influence the association between SEP and DNAm?

The majority of studies covered in this review examined a single life-course period of SEP exposure in relation to DNAm. However, nine studies—two global [46, 47], two candidate [59, 61], and five EWAS [69, 71, 72, 75, 77]—investigated the timing and/or duration of SEP by either capturing more than one life-course period of SEP (e.g., child and adult SEP) or analyzing more than one timepoint of SEP exposure within the same life-course period (e.g., captured child SEP at three different assessments to compare between very early, early, and middle childhood). These studies found evidence that the timing and duration of SEP may influence the association between SEP and DNAm (Table 4), although the magnitude and direction of these timing effects were inconsistent across studies.

**Table 4:** Findings from nine studies investigating the timing and/or duration of socioeconomic position and DNA methylation

References	DNAm approach	SEP exposure age(s)^a^	Reported age(s) at DNAm^b^	Key findings^c^
*Sensitive period of child SEP on child or adult DNAm*				
Dunn et al. [71]	EWAS	Child (prospectively measured 5×)	7	Neighborhood disadvantage and financial stress associated with 10 and 9 CpGs (*p*_bonf_ < 1 × 10^−7^), respectively, with 8 and 2 of these differentially methylated sites relating to the developmental timing of SEP exposure in very early childhood (birth to age 2)
Lam et al. [72]	EWAS	Child (retrospectively measured) + Adult	Mdn = 33.04	Low child SEP associated with 3 CpGs (*q* < 0.25), while current (adult) SEP did not
Borghol et al. [69]	EWAS	Child (prospectively measured) + Adult	42 + 45	Child SEP associated with 1252 gene promoters, while adult SEP associated with 545 (*q* ≤ 0.2). Child and adult SEP-associated promoter sets overlapped at 63 promoters
*Sensitive period of adult SEP on adult DNAm*				
Stringhini et al. [61]	Candidate Gene	Child (retrospectively measured) + Adult	~ 45–63	Current (adult) SEP associated with 41 CpGs in 10 genes (*q* ≤ 5.10 × 10^−3^), while child SEP did not have any associations when FDR procedure was applied
Subramanyam et al. [46]	Global DNAm	Child (retrospectively measured) + Adult	M = 61	One SD higher adult wealth associated, on average, with 0.09% higher Alu (*p* < 0.01) and 0.15% lower LINE-1 DNAm (*p* < 0.01), while adult income, adult education, and child SEP did not associate with LINE-1 or Alu DNAm
*Sensitive periods of both child and adult SEP on adult DNAm*				
Tehranifar et al. [47]	Global DNAm	Child (prospectively measured) + Adult	M = 43	For child SEP, lower maternal education and lower family income associated with higher mean levels of Sat-2 DNAm (*p* < 0.05 and *p* < 0.10, respectively); single-parent family structure compared to two-parent structure associated with higher Alu methylation (*p* < 0.05). Adult income associated with increased LINE-1 methylation (*p* < 0.05)
Suderman et al. [77]	EWAS	Child (prospectively measured) + Adult	45	Child SEP associated with 2 CpGs, while adult SEP associated with 3 CpGs (*q* < 0.2) in whole blood or LCLs. None of the SEP-associated CpGs were shared between child and adult SEP
Needham et al. [59]	Candidate Gene	Child (retrospectively measured) + Adult	M = 69.55	Low child SEP associated with DNAm in 3 stress- and 2 inflammation-related genes, whereas low adult SEP primarily associated with DNAm in inflammation-related genes (5 inflammation- and 1 stress-related gene; all *p* < 0.05; *q* < 0.20). SEP-DNAm associations between child and adult SEP overlapped at 3 of 9 total candidate genes
*Effects of life-course SEP trajectories on adult DNAm*				
McDade et al. [75]	EWAS	Child + Adult (prospectively measured 4×)	M = 20.9	Persistently low SEP from infancy to young adulthood associated with 2546 CpGs (*q* < 0.05), compared to persistently high SEP. One CpG associated with DNAm for upward SEP mobility group and no sites associated with the downward mobility group (*q* < 0.05)
Stringhini et al. [61]	Candidate Gene	Child (retrospectively measured) + Adult	~ 45–63	Persistently low SEP from childhood to adulthood associated with 12 CpGs in 6 genes, compared to persistently high SEP, while downward SEP and upward SEP mobility groups associated with 5 CpGs in 4 genes and 1 CpG in 1 gene, respectively (*q* ≤ 1.49 × 10^−3^)
Needham et al. [59]	Candidate Gene	Child (retrospectively measured) + Adult	M = 69.55	Persistently low SEP from childhood to adulthood associated with DNAm in 5 genes, upward SEP mobility associated with 1 gene, and downward mobility associated with 2 genes in comparison with persistently high SEP (*p* < 0.05; *q* < 0.20)

Studies presented in this table are categorized by general findings in support of sensitive periods of child SEP, adult SEP, or both and DNAm, and by findings from SEP trajectory studies. Within these categories, studies are shown in descending order of DNAm assessment age

*CpGs* CpG site, *DNAm* DNA methylation, *FDR* false discovery rate, *LCLs* lymphoblastoid cell lines, *M* mean, *Mdn* median, *SEP* socioeconomic position, *SD* standard deviation

^a^SEP exposure age column reports the life-course period of exposure captured (prenatal, child, adult) and notes whether measure was prospectively or retrospectively assessed. Number of assessments indicated (e.g., 2×, 3×) if same SEP indicator was measured at more than one timepoint per life course group

^b^Reported age at DNAm age column reports the age of DNAm assessment in years (as reported by individual studies)

^c^*p* values indicate uncorrected significance thresholds; *q*-values and *p*_bonf_-values indicate *p* values after adjustment for multiple testing by false discovery rate (FDR) and Bonferroni methods, respectively

With respect to the relative importance of exposure timing, two [69, 72] of seven studies comparing child and adult SEP found stronger support for sensitive periods of child SEP with adult DNAm differences. By contrast, two other studies [46, 61] found stronger associations between adult SEP and adult DNAm differences compared to child SEP, noting that the lack of associations for child SEP may be due to measurement limitations (e.g., retrospectively assessed, limited SEP variability). The remaining three studies [47, 59, 77] found support for both child and adult SEP associations with adult DNAm differences, observing diverse DNAm changes between child and adult indicators. Finally, one study [71] analyzed indicators of low SEP measured repeatedly across different childhood periods. Findings from that study suggested sensitive-period effects, such that low SEP in very early childhood (before age 3) was associated with 10 of 19 differentially methylated CpGs at age 7 (*p* < 1 × 10^−7^).

Three studies in this group [59, 61, 75] also captured effects of life-course SEP trajectories on adult DNAm, such as moving from low child to high adult SEP (Table 4). These studies consistently found more DNAm differences between persistently low SEP (low child and adult SEP) and persistently high SEP groups, with fewer or no DNAm differences observed for comparisons between either upward or downward mobility groups (moving from low child SEP to high adult SEP, or vice versa) and individuals with persistently high SEP. Findings between the upward and downward mobility groups were inconsistent across studies.

Taken together, these findings provide evidence for an effect of SEP timing and duration on DNAm, with early evidence suggesting that this relationship may be unique for SEP indicators measured in childhood and persistent exposure to low SEP across the life course.

### (4) Do different SEP indicators associate differently with DNAm profiles?

**Fig. 3:**
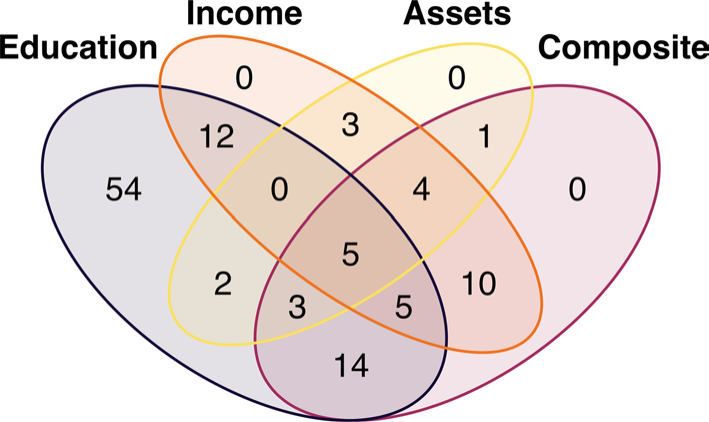
Venn diagram illustrating the overlap of unique, top CpG sites across socioeconomic position domains. Pattern of overlap in 113 significant socioeconomic position (SEP)-associated CpGs (FDR < 0.05) appearing in more than one study across four SEP domains: education, income, assets (household), and composite. As shown for income, there were no unique associations found among the 39 CpGs predicted by income, with 12 CpGs overlapping with education, 10 with composite, 3 with assets, and the remaining 14 overlapping with two or more domains. CpG-level data were compiled from summary statistics of nine epigenome-wide association studies utilizing the Illumina Human Methylation 450k array. For more information on how these summary statistics were derived, see Additional file 1. For a list of 113 associated CpG IDs, see Additional file 2: Table S5

We addressed our fourth research question in two parts, using summary statistics compiled from the nine EWAS studies that used the 450k array [68, 70, 71, 73–75, 77–79] (Additional file 2: Tables S4). First, we applied our own significance threshold to study-level summary statistics (FDR < 0.05) to identify unique, top CpGs present in two or more studies; using this approach, we found 113 unique CpGs, with five CpGs appearing between three different studies. Within the same SEP domain, 14 of the 113 unique CpGs appeared across more than one study: 12 within the education domain, followed by one for income and one for composite (Additional file 2: Tables S5). These 113 unique CpGs spanned 264 total associations between SEP and DNAm across domains; education had the highest number of associations (*n* = 95), of which 54 (57%) were unique loci (Fig. 3). Only five CpGs were associated across all domains. These findings suggest that while some SEP-related DNAm signals may replicate across studies, no underlying pathways or loci consistently emerge from the current literature.

**Fig. 4:**
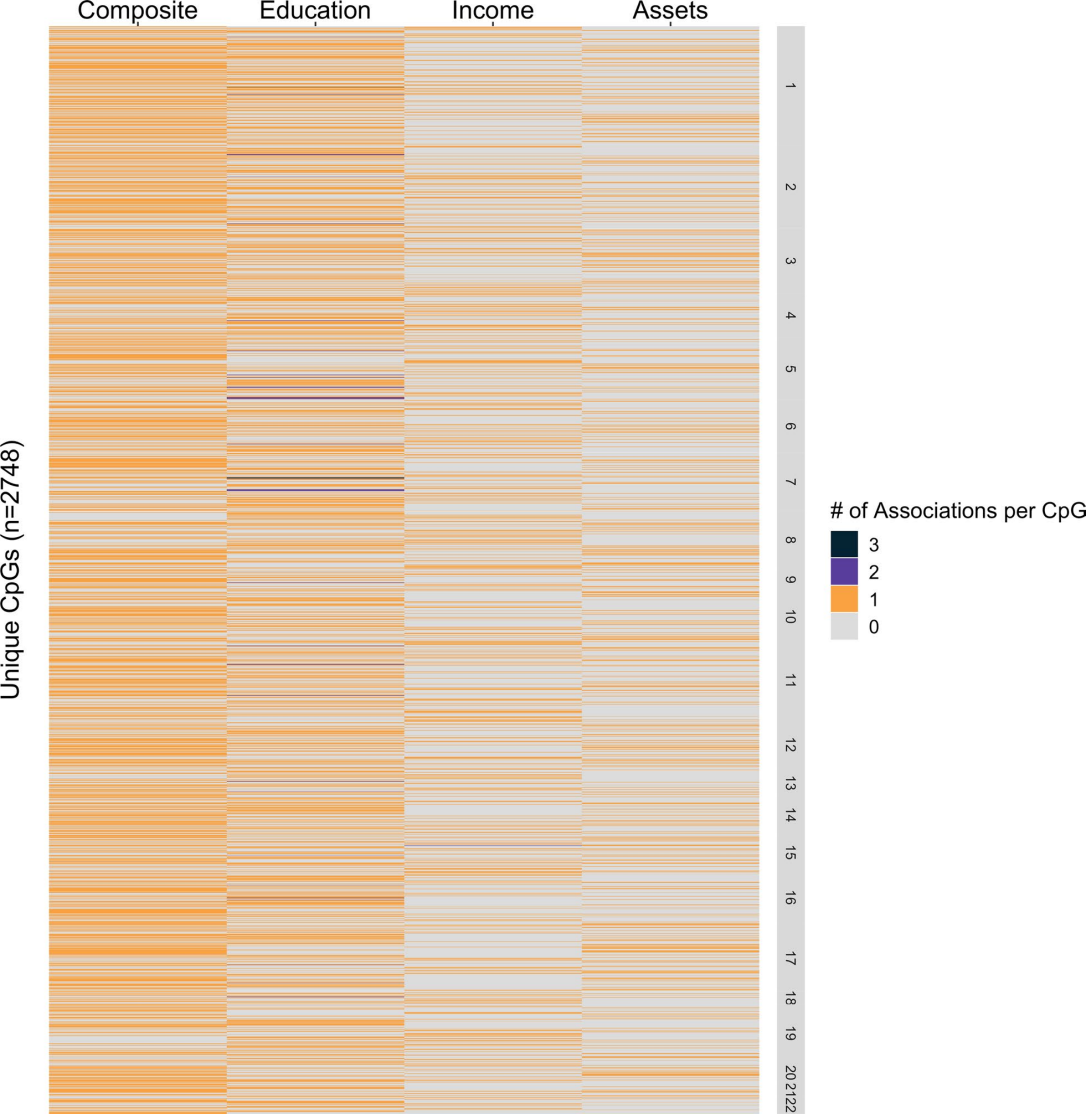
Heat map demonstrating the overlap of shared, top CpG sites across socioeconomic position domains. The CpGs associated (FDR < 0.05) with four socioeconomic position (SEP) domains, composite, education, income, and assets (household), are shown here. We adjusted for false discovery rate (FDR) within eight epigenome-wide association studies using individual CpG-level summary statistics, including only CpGs analyzed across all studies, arriving at 2748 unique CpGs across six studies (FDR < 0.05). Colors indicate the number of associations per CpG per SEP domain, ranging from 0 to 3. For each SEP domain, a CpG received a value of 0 if it did not survive FDR adjustment or was not analyzed in that domain. Individual CpGs were ordered along the y-axis by chromosomal position, though no apparent pattern in chromosomal position was identified. In total, 59 CpGs appeared in two different studies and 5 CpGs appeared in three different studies. For associations shared between more than one study in each column, 36 CpGs associated with education between two studies and 3 CpGs associated with education between three studies. In the income domain, one CpG associated between two different studies. No CpGs were shared between studies for composite and assets. Composite measures associated with the highest number of CpGs (*n* = 1389), followed by education (*n* = 1156), income (*n* = 624), and assets (*n* = 544). There was little overlap in CpGs between domains, with 88% of CpGs in the income domain having unique signal, followed by 62% for education, 47% for composite, and 21% for assets. See Additional file 1: Table S4 for more details on summary statistics

Because studies did not always assess the same loci, we performed a second set of analyses to determine the level of overlap in top DNAm signals across SEP domains among CpGs tested in all nine studies. Here, we filtered the summary statistics to include only CpGs analyzed across all studies (*n* = 53 808). After applying an FDR adjustment, 3670 CpG associations remained (FDR < 0.05), of which more than half (*n* = 2002; 55%) were unique to a single SEP domain (Fig. 4). Composite measures were linked to the highest total number of significant CpGs (1389), 652 of which (47%) were unique. Education was associated with the second highest total number of CpGs (1114), 686 of which (62%) were unique. A total of 623 associated CpGs were reported for income and 544 for assets, of which 548 (88%) and 116 (21%) were unique, respectively. Overall, these results suggest that different SEP indicators, particularly education and income, may represent distinct aspects of the socioeconomic environment and thereby present unique relationships with DNAm.

## Discussion

Four main findings emerged from this review. First, indicators of child and adult SEP shared little overlap in adult DNAm profiles, suggesting that SEP may become biologically embedded through distinct and potentially time-dependent pathways across development. Such findings are consistent with prior life-course research showing that risks for adverse health outcomes differentially arise from child and adult SEP [80]. For example, behavioral and health risk factors (e.g., cigarette smoking, low exercise levels) are more strongly linked to adult SEP, while physiological risk factors (e.g., BMI, cardiovascular diseases) are more strongly associated with child SEP [81]. However, less than 25% of studies included in the present review directly compared the associations from both child and adult SEP with DNAm differences in adulthood. In addition, nearly half of these studies captured child SEP *retrospectively* during adulthood, with all measuring DNAm cross-sectionally in adulthood. Although these studies offer preliminary information on how SEP across the life course associates with DNAm, study findings are subject to inherent design limitations, such as potential bias of retrospective reports [82]. Additionally, studies investigating both child and adult SEP did not account for familial or prior SEP (e.g., SEP assessed through parental measures) when investigating current or adult exposure status; however, because child and adult SEP are often highly intertwined [83], future studies should investigate whether controlling for familial effects influences the relationship between SEP and DNAm across the life course. Building from these findings, longitudinal, birth cohort data are needed to analyze *prospective* SEP measures and repeated DNAm measures to determine whether differences in DNAm appear early in life, later in adulthood, and/or change over the life course.

Second, suggestive evidence emerged for SEP timing and duration effects on DNAm, consistent with life-course theories on mobility [84, 85], sensitive periods [86, 87], and accumulation of risk effects [88, 89]. In particular, three trajectory studies evaluated mobility, finding most differences in DNAm profiles for groups exposed to persistently low compared to persistently high SEP in childhood and adulthood. These findings are consistent with prior studies showing cumulative effects of socioeconomic disadvantage on poor health outcomes into adulthood [80, 90, 91]. Of note, findings from these trajectory studies also showed that compared to persistently high SEP, upward/downward SEP mobility resulted in fewer DNAm differences than persistently low SEP. These findings suggest that early-life DNAm patterns may not be fixed in development, but rather SEP effects might be modified through *changes* in SEP later in life [83, 92]. Additionally, one study tested a sensitive period hypothesis at multiple stages in childhood, showing that SEP captured in the first 3 years of life, as compared to later developmental periods, was the strongest predictor of DNAm differences at age 7 [71].

Third, we found little overlap in DNAm patterns across SEP domains, suggesting that different SEP indicators likely represent different aspects of the socioeconomic environment, and thus, may leave distinct biological signatures. Past reviews have examined the relationship between various SEP indicators and health outcomes, noting that SEP indicators are independent from each other and that measures such as education and income are not interchangeable [93, 94]. Yet, in the current review, education and income were most commonly investigated across studies, leaving other key SEP indicators such as neighborhood-level SEP relatively absent in the broader epigenetic literature, despite their effects on numerous health outcomes [95, 96]. Additionally, accumulating evidence suggests *perceptions* of SEP may have differential associations with behavioral/health outcomes compared to more *objective* SEP measures [97], and in some cases, perceived experiences of adversity may influence subsequent neurobiology more than objective features of the experience itself [98]. However, only five studies (14%) evaluated both objective and subjective SEP indicators (e.g., self-reported neighborhood quality versus census-tract-level variables of neighborhood disadvantage) in the current review. These gaps in SEP measurement highlight the need for future epigenetic studies to more evenly capture SEP operating across different socioecological levels, domains, and data collection methods, in order to elucidate the potentially different downstream health effects of various SEP exposures [99–101].

Finally, our review made clear the overall paucity of life-course study designs in the current literature, the widespread heterogeneity that exists between SEP-DNAm studies, the mismatch of SEP measurements to the target population studied, and the general under-representation of more diverse samples. Despite assessing similar SEP constructs, there was little consensus in how studies actually measured SEP, with over 40 different operational definitions included. Additionally, most studies relied on different dimensions of common SEP measures, which are not necessarily optimized for the study of SEP across different demographic groups. For instance, measures of overall wealth in adults—defined as the total value of all physical and financial assets, such as homes, vehicles, investments, and saving accounts [102]—are more stable indicators of SEP and health differences across the adult life course than commonly used measures, such as income [103, 104]. However, only one [46] of 17 studies studying adults measured wealth; most adult studies instead measured SEP via education, which fails to capture assets such as housing, car ownership, and investments [5]. Moreover, 27% of studies did not explicitly report race/ethnicity sample characteristics, even though these factors can greatly influence experiences and effects of SEP [34, 105, 106]. As such, future studies should control for these potential race/ethnicity differences, as well as include, when possible, methodologies that account for genetic variation, as allelic differences can influence DNAm (e.g., methylation quantitative trait loci [107]; principal components of genetic background [108]; etc.). Without greater consensus on best practices in defining and reporting SEP [96, 109], and testing these associations in diverse sociodemographic samples [110], comparisons between outcomes will remain challenging to interpret, and potential differences across racial/ethnic identities and other demographics factors will be difficult to discern.

### Future directions

Given these findings, how should the field move forward to build a next generation of robust and well-designed studies to study SEP-DNAm associations? We provide four recommendations to facilitate a clearer picture of not only *whether*, but also *when* and *how* different aspects of the socioeconomic environment influence DNAm and broader biological processes. Given the rising number of SEP-DNAm studies with disparate SEP indicators and DNAm methods, the growing availability of epigenome-wide technologies, and the political attention to the impacts of socioeconomic inequality, now is the time for more rigorous studies to characterize SEP effects on DNAm outcomes.

First, the field needs to design studies that allow for stronger characterization of the causal links between SEP and DNAm across the life course. We think this can be achieved in at least two ways. For one, longitudinal datasets—containing repeated SEP and DNAm measures collected across time—are key to strengthening causal inference in observational research [111]. As we showed, less than a quarter of studies in the current review adopted longitudinal study designs capable of testing the causal and time-dependent effects of SEP on DNAm. With existing and emerging longitudinal datasets, researchers can apply novel life-course statistical modeling [112] and causal mediation [113] approaches to explore underlying exposure–outcome relationships in the high-dimensional epigenetics settings [114]. For example, Mendelian randomization, a technique to reduce potential confounding and reverse causality in observational studies [115], allows researchers to leverage genetic data to tease apart underlying causal relationships between SEP and DNAm. Moreover, experimental study designs offer key opportunities to strengthen the evidence based on the biological embedding of SEP, while overcoming potential confounding effects present in observational research. For instance, Baby’s First Years, an ongoing randomized control study evaluating the impact of monthly unconditional cash gifts to low-income mothers [116], incorporates biomarkers that allow for greater probing of the effects of socioeconomic disadvantage on different neurobiological processes. Experimental study designs have also been extended to epigenetic outcomes and can be used to identify health interventions that shift or reverse DNAm differences [117]. By using more rigorous observational and experimental designs as we summarized, the field will be better positioned to identify the causal pathways underlying the biological embedding of SEP, understand the effects of DNAm on health more directly, and use such insights to drive economic policies and other interventions.

Second, studies should prioritize samples collected from (a) lower-SEP countries, (b) broad SEP gradients, and (c) diverse racial/ethnic identities, to determine whether more striking SEP-DNAm associations are present between larger contrasts of SEP and diverse population subgroups. When interpreting results, researchers should also consider country/state-level societal factors, such as health care (e.g., access/barriers to care) and education policies (e.g., free school meals), that may modulate SEP’s impact on health/behavioral outcomes [118–120].

Third, beyond including repeated DNAm assessments in future studies, epigenetic analyses should also be thoughtful around approaches to DNAm analysis, tissue specificity, multiple testing procedures, covariates, reporting of results, and replication/validation efforts [121, 122]. Because most SEP and DNAm studies originate from samples initially designed to test other associations, they will often be limited in ways that can only be reconciled using statistical methods or careful considerations of confounding effects. In addition to building SEP-DNAm studies principally designed for such purposes, we also recommend that researchers reference established guidelines for the reporting and analysis of observational studies (i.e., STROBE) [123–125] during the conceptual design phase of their study, which will help improve the overall reproducibility and consistency of associations between future epigenetic studies, even across diverse datasets.

Fourth, it is crucial that researchers more precisely conceptualize and measure SEP, which can be achieved by (a) selecting SEP variables that represent different levels of SEP in a given population (e.g., indicators of wealth should be prioritized in elderly populations); (b) employing consistent terminology to define the components captured by SEP measures, referenced through glossaries [1, 3, 126], national/institutional recommendations [127, 128], and prior studies; and (c) analyzing a comprehensive set of SEP indicators (i.e., across different domains, levels, and collection methods), including the individual components of composite measures. The following studies may be helpful examples for assessing SEP indicators operating at *different* levels [42, 71] or capturing both objective and subjective indicators at the *same* level [60, 73]. Furthermore, prior research has shown that individuals with low SEP experience more frequent stressful life events and report more psychological distress than their higher SEP counterparts [129, 130]. Future research investigating how other psychological stressors associated with SEP mediate the SEP-DNAm relationship will help to further untangle how SEP ultimately gets under the skin to influence health outcomes.

### Limitations

Although this review offers a comprehensive overview on the state of the SEP-DNAm literature, there are several limitations of the review process worth noting. First, while we covered three major types of DNAm approaches (i.e., global, candidate, EWAS), we excluded other approaches that did not examine direct measures of DNAm levels. In particular, we excluded studies of the epigenetic clock, as they measure *biological aging* estimated through DNAm [131], rather than DNAm *levels*. Although prior studies have shown that socioeconomic disadvantage has been linked to accelerated epigenetic aging in multiple empirical studies [132, 133], the present review focuses on specific DNAm changes associated with SEP, rather than the composite measure of aging and health described by epigenetic clocks. Nevertheless, future studies should continue to interrogate the impact of SEP on epigenetic age, as they can provide insight into the broader effects of SEP on human health and aging.

Second, given our broad inclusion criteria, we included studies measuring DNAm using different tissue types and, thus, comparisons across study findings should be interpreted with caution as DNAm is known to be tissue- and/or cell-type specific [134, 135]. Although prior studies comparing DNAm between tissues associated with certain clinical phenotypes have found that some loci display high correlations between peripheral and central tissues [121, 136, 137], it remains relatively unknown how DNAm patterns across tissue types associate with complex social and environmental constructs like SEP. Future epigenetic studies on SEP should, when possible, assess DNAm correlation between tissue types within the same sample to reliably identify either cross-tissue or within-tissue high-risk biomarkers. However, researchers must carefully consider their study design and research questions to adequately address issues of tissue concordance and specificity. For example, epigenetic studies interested in psychiatric or neurological outcomes should ideally analyze brain tissue, and, if not available, a tissue that is highly correlated with brain tissue for DNAm. Another example is whether a study is interested in establishing diagnostic performance of DNAm biomarkers for a certain disease. In this scenario, researchers may want to compare several different types of surrogate tissue samples within the same individuals to establish the validity of risk prediction in easily accessible tissues.

Third, all studies included in the review were observational in nature, with data assessed either cross-sectionally or prospectively. Therefore, study findings only suggest a link, rather than a causal effect of SEP on DNAm levels. The strengths and limitations of the DNAm approaches (global, candidate, EWAS) should also be considered when interpreting individual study findings. For instance, candidate gene studies are difficult to replicate, as findings are often influenced by the number of candidate genes targeted [25]. By contrast, epigenome-wide analyses are not biased by the selection of target genes but might be underpowered in some instances to detect subtle changes to epigenomic patterns [26]. Furthermore, the SEP indicators were not standardized within and between studies (i.e., measurement bias), limiting their interpretability and power to detect consistent and reliable associations with DNAm.

Finally, no formal quality assessment or meta-analysis was performed on these data. However, this scoping review, which maps the current state of evidence and proposes promising next steps for the field, serves as a steppingstone for future systematic and meta-analytic reports on the topic, once between-study heterogeneity is reduced.

## Conclusion

As socioeconomic inequality continues to grow on a global scale [138], the health consequences of SEP and its correlates increase worldwide. Because SEP is a fundamental social determinant, influencing nearly all aspects of the environment that contribute to overall health, it must be considered in epigenetic studies of social and behavioral traits [139], whether as a control or independent variable. To better understand how the socioeconomic environment interacts with the epigenome and other biological processes to contribute to health disparities, researchers must also consider the implications and limitations of evidence due to the diversity of SEP measures, while also applying rigorous design and analytic approaches that allow for the investigation of SEP timing, duration, and type. With these efforts, we can tackle the complexities of how SEP becomes biologically embedded and help guide future intervention and prevention strategies to effectively reduce SEP-related health disparities across diverse populations.

## Supplementary Information

**Additional file 1:** Supplemental methods, results, and figures.

**Additional file 2:** Supplemental tables.

## Data Availability

All data collected and analyzed in the present study are included in this published article, its additional files, or are available upon request from the corresponding author.

## Abbreviations

CpGs: Cytosine-phosphate-guanine site
DNAm: DNA methylation
EWAS: Epigenome-wide association study
SEP: Socioeconomic position
