# Supplementary material for: Associations between indicators of socioeconomic position and DNA methylation: a scoping review
Source: Clin Epigenetics. 2021 Dec 14;13:221. doi: 10.1186/s13148-021-01189-0 (PMC8672601; doi:10.1186/s13148-021-01189-0)
Supplement: Supplementary file 1 — Additional file 1: Supplemental methods, results, and figures. [file 13148_2021_1189_MOESM1_ESM.docx]

**Additional file 1**

Study identification

*Full search terms*

We systematically searched for articles published from inception through September 18, 2019 on PubMed and PsycINFO by using a combination of index/MeSH terms (i.e., keywords) and individual terms located in the title or abstract that were related to socioeconomic position (SEP) and DNA methylation (DNAm). To capture the multifaceted nature of SEP, we included terms for the following domains: a) education b) occupation (including employment), c) income (including financial difficulties), and d) neighborhood characteristics. No language, publication date/status, or age restrictions were imposed. The following were the final search terms used to search for articles in PubMed: *(“Socioeconomic Factors”[Mesh] OR “Income”[Mesh:NoExp] OR Income[tiab] OR Income distribution[tiab] OR Living standard*[tiab] OR Standard of living[tiab] OR Socioeconomic ­­status*[tiab] OR Socioeconomic factor*[tiab] OR Occupation[tiab] OR Vocation[tiab] OR Social class*[tiab] OR Poverty area*[tiab] OR Neighborhood disadvantage[tiab] OR Neighborhood characteristics[tiab] OR adversity[tiab]) AND (“DNA Methylation”[Mesh] OR “Epigenesis, Genetic”[Mesh] OR “Epigenomics”[Mesh] OR DNA Methylation*[tiab] OR Epigenet*[tiab] OR Epigenetic Process*[tiab] OR Genetic epigenesis[tiab]).* Terms followed by “[Mesh]” were index terms used to search by subject content of indexed journal articles. Individual terms followed by “[tiab]” searched for articles that included the term in the abstract or the title; * denotes truncation.

In PsycINFO database, the following were the final search terms used to search for articles in PsycINFO: *((DE "Socioeconomic Status" OR DE "Family Socioeconomic Level" OR DE "Income Level" DE "Social Class"OR DE "Parental Occupation" OR DE "Occupational Status" OR DE "Occupational Mobility" OR DE "Occupations" OR DE "Occupational Success"OR DE "Poverty" OR DE "Social Deprivation" OR DE “Disadvantaged” OR DE “Poverty” OR DE “Income (Economic)” OR DE “Income Level” OR DE “Financial Strain” OR DE "Educational Attainment Level") OR TI (“social class” OR “socioeconomic*” OR “income” OR “occupation” OR “education”) OR AB (“social class” OR “socioeconomic*” OR “income” OR “occupation” OR “education”) OR SU “Socioeconomic Status”)) AND (DE “Epigenetics” OR TI (“DNA Methylation*” OR “epigenetics” OR “methylation”) OR SU “Epigenetics” OR AB (“DNA Methylation*” OR “epigenetics” OR “methylation”)).* Terms following “DE” and “SU” were index terms used to search by subject content of indexed journal articles and by subject heading; individual terms following “TI” and “AB” searched for articles that included the term in the abstract or the title; * denotes truncation.

Study selection

*Inclusion and exclusion criteria*

The search and selection procedure is shown in **Figure 1.** The PubMed and PsycINFO search returned 478 results, which were exported into EndNote for evaluation. Titles and abstracts were reviewed using the following inclusion criterion: human empirical studies that examined an independent association between at least one SEP exposure measure and DNAm as an outcome. Most articles (366) were excluded because they did not include an SEP measure as an exposure and/or did not include DNAm as an outcome. Thirteen articles were removed because they measured DNAm with an epigenetic biomarker of age to estimate “epigenetic age,” or estimates of biological age based on DNAm. Although epigenetic clock papers extract DNAm with similar tools/arrays as other DNAm studies, the mechanism of interest in these studies is specifically *biological aging.* Extensive reviews on the topic of biological aging have been previously conducted (4-6), and it is well established that accelerated biological aging measured via DNAm is linked to adverse health outcomes (2, 3). By contrast, it is unknown whether decreased or increased DNAm *levels* at different loci across the genome are adaptive or maladaptive to health. Given these differences, we focused our review on DNAm levels as the main outcome of interest rather than other epigenetic processes in the literature. Another 10 were removed because they did not include a healthy control group or their sample was homogenous for SEP status (e.g., entirely low income). Four animal studies were removed. Three studies were excluded because they combined SEP and non-SEP (e.g., childhood abuse or parenting stress) measures into one aggregated composite measure. Fifty-five were reviews, overviews, or commentaries and were also excluded. We identified six additional studies by reviewing reference lists of 29 eligible publications and also added two known publications to our review. In final, 37 studies were included in this review.

Data extraction and evaluation

*SEP sample features*

To assess the range (spectrum) of SEP across studies in the current review, we characterized the level of SEP severity (i.e., very low, low, or mid/high) in each individual study sample by the following criteria:

1. For *very low* SEP, sample included participants who were either (a) living below poverty threshold line, assessed by census-tract data, (b) eligible or on a form of public assistance, including public health insurance, housing or food subsidies, or (c) living in a country outside of a WEIRD setting (i.e., sample drawn from populations outside of White, Educated, Industrialized, Rich and Democratic countries) (6).
2. For *low* SEP, study coded participants as low-high SEP for at least two distinct SEP indicators (e.g., education and income).
3. For *mid/high* SEP, the above criteria were not met (e.g., sample SEP descriptives were only reported for one SEP indicator).

Based on the above criteria, 21 studies qualified as very low or low SEP, with the remaining 16 having mid/high SEP samples. The range of SEP in a given study sample was considered *wide* (i.e., very low or low to mid/high SEP) if 20-50% of participants qualified for very low/low SEP. Although the majority of studies in the current review captured very low/low SEP participants, only 12 study samples (32%) covered a wide SEP range. If a study sample consisted of 80% (or more) mid/high SEP participants or if the sample was entirely mid/high SEP, it was classified as having a *small* SEP range (17 studies; 46%). A total of eight studies did not fully report the percentages of SEP levels and thus SEP severity/range could not be assessed. See **Additional file 2: Tables S1–S3** for study-level SEP sample ranges.

*SEP exposure features*

The following domains were used to categorize each measure indicating a component of the SEP construct: education, occupation, income, neighborhood, subsidy, composite, and other. All measures relating to *education* (e.g., highest level of education, number of years of schooling, highest degree, etc.) were included in the education domain. Measures related to occupation or employment (e.g., highest level of household occupation, unemployment) were included in the occupation domain. Measures related to *income* (e.g., weekly or annual income) were included in the income domain. Measures of the *neighborhood* domain captured physical, structural, and functional aspects of an individual’s neighborhood through either self-reported questionnaires or by geography/zip code via census-tract data (7). The *subsidy* domain included measures indicating whether individuals were eligible or on a form of public assistance, such as food or housing subsidies. We also created a domain for *composite* measures*,* which included studies that used measures that were either: a) a cumulative score derived by aggregating multiple scores from indicators that spanned more than one domain of SEP (summing, averaging, standardizing, etc.), or b) from pre-existing scales or indexes measuring multiple domains of SEP (e.g., Hollingshead Four Factor Index of Socioeconomic Status). Finally, the *other* domain included miscellaneous indicators of SEP, such as poverty status, marital status, crowded dwelling, and difficulty affording household assets.

Ages of SEP exposure were grouped by life-course period: prenatal (<0), birth (0), child (0-18 years), adult (18+ years). Studies that captured SEP spanning early life to adulthood (i.e., prenatal, birth, or child to adult) were classified as “life course” for exposure age. Method of collection was grouped into five categories: caregiver report, self-report, multigenerational (i.e., both caregiver and self-report), census-tract (i.e., taken from government official statistical subdivisions of a county of geographical equivalent), and cohort-level summary statistics. For longitudinal and prospective studies, SEP exposures were also reported as prospective or retrospective for method of collection. Scale of measurement defined how each measure was statistically encoded for analysis, grouped by continuous (continuous numerical scale), dichotomous (binary), categorical (unranked), and ordinal (ranked). For more details on individual study SEP indicators and definitions, see **Additional file 2: Tables S1–S3**.

Summary statistics analyses and Additional file results

We addressed our fourth research question (do different SEP indicators show differential DNAm profiles?) in two parts with compiled summary statistics of the nine epigenome-wide studies (EWAS) included in the current review that used the Illumina Human Methylation 450k array method (8-16). Of note, we contacted authors of seven studies for these summary statistics and the remaining two were retrieved online (12, 14). We adjusted *p*-values using a 5% false-discovery rate (FDR) (17) within each study by each SEP indicator analyzed and annotated CpG sites (CpGs) to the nearest gene (located in the gene body or within 300 kb of a transcription start site, TSS) and chromosome position using the *FDb.InfiniumMethylation.hg19* package in R/Bioconductor. Additional descriptives of the summary statistics by individual study level, including sample size, age at assessments, tissue type(s), and covariates are included in **Additional file 2: Table S4**. In part one, we compared top CpGs (FDR < 0.05) between the nine studies. Of note, one of these nine studies (8) was a mediation analysis and only the top CpGs included in the mediator were available for analysis. Before FDR adjustment, there were a total of 482 674 unique CpGs spanning seven domains: composite, education, household assets, income, neighborhood, occupation, and other. A total of 7652 unique CpGs survived adjustment for FDR < 0.05 across eight studies and four SEP domains (i.e., composite, education, income, household assets). We found 113 significant CpGs appearing in more than one study, with five appearing between three studies and the remaining between two studies (see **Additional file 2: Table S5** for list of individual CpG IDs and annotated genes).

These 113 CpGs had 264 associations across four domains of SEP. The majority of associations were found for education (95 total), followed by composite (42), income (39), and assets (18; **Figure 3**). As indicated in **Additional file 2: Table S5,** over half (n=67; 59%) of these 113 CpGs appeared within the same SEP domain across more than one study. However, nearly half (n=53; 47%) of CpGs appeared solely between the two meta-analyses on educational attainment (12, 15), which shared partially overlapping samples. Eight of these 53 sites were previously reported as shared between these two meta-analyses, for adjusted models additionally controlling for smoking (15). For the remaining 14 CpGs that overlapped across one or more studies with different samples, 12 CpGs appeared within the education domain, followed by one for income and one for composite. In total, CpGs annotated to 73 unique genes, with *AHRR* annotating to the most unique number of CpGs (n=9), followed by *CDK6*, *MYO1G*, and *PRSS23* annotating to four total CpGs each.

In part two, we performed a between-study comparison of all analyzed CpGs predicted by SEP at FDR < 0.05 and assessed the extent of overlap in top CpGs between SEP domains. We excluded CpGs from the aforementioned mediation analysis and filtered the compiled summary statistics to (a) only CpGs analyzed across all eight studies, and (b) CpGs that survived FDR < 0.05 adjustment. Through these two filters we identified 2748 unique CpGs between six studies (9, 11-13, 15, 16) spanning four SEP domains (i.e., composite, education, income, and household assets). Of note, two of the six studies included in this analysis analyzed composite measures based on indicators of education, income, and/or assets included in **Figure 4**, which may explain the lack of unique signal found for composite and assets.

**References**

1. Colich NL, Rosen ML, Williams ES, McLaughlin KA. Biological aging in childhood and adolescence following experiences of threat and deprivation: A systematic review and meta-analysis. Psychol Bull. 2020;146(9):721-64.

2. Jones MJ, Goodman SJ, Kobor MS. DNA methylation and healthy human aging. Aging Cell. 2015;14(6):924-32.

3. Oblak L, van der Zaag J, Higgins-Chen AT, Levine ME, Boks MP. A systematic review of biological, social and environmental factors associated with epigenetic clock acceleration. Ageing research reviews. 2021;69:101348.

4. Steptoe A, Zaninotto P. Lower socioeconomic status and the acceleration of aging: An outcome-wide analysis. Proceedings of the National Academy of Sciences. 2020;117(26):14911-7.

5. Austin MK, Chen E, Ross KM, McEwen LM, Maclsaac JL, Kobor MS, et al. Early-life socioeconomic disadvantage, not current, predicts accelerated epigenetic aging of monocytes. Psychoneuroendocrinology. 2018;97:131-4.

6. Henrich J, Heine SJ, Norenzayan A. The weirdest people in the world? Behavioral and Brain Sciences. 2010;33(2-3):61-83.

7. Cutrona CE, Wallace G, Wesner KA. Neighborhood Characteristics and Depression:An Examination of Stress Processes. Current Directions in Psychological Science. 2006;15(4):188-92.

8. Beach SR, Lei MK, Brody GH, Kim S, Barton AW, Dogan MV, et al. Parenting, Socioeconomic Status Risk, and Later Young Adult Health: Exploration of Opposing Indirect Effects via DNA Methylation. Child Dev. 2016;87(1):111-21.

9. Bush NR, Edgar RD, Park M, MacIsaac JL, McEwen LM, Adler NE, et al. The biological embedding of early-life socioeconomic status and family adversity in children's genome-wide DNA methylation. Epigenomics. 2018.

10. Dunn EC, Soare TW, Zhu Y, Simpkin AJ, Suderman MJ, Klengel T, et al. Sensitive periods for the effect of childhood adversity on DNA methylation: results from a prospective, longitudinal study. Biological Psychiatry. 2019;85(10):838-49.

11. Laubach ZM, Perng W, Cardenas A, Rifas-Shiman SL, Oken E, DeMeo D, et al. Socioeconomic status and DNA methylation from birth through mid-childhood: a prospective study in Project Viva. Epigenomics. 2019;11(12):1413-27.

12. Karlsson Linnér R, Marioni RE, Rietveld CA, Simpkin AJ, Davies NM, Watanabe K, et al. An epigenome-wide association study meta-analysis of educational attainment. Mol Psychiatry. 2017;22(12):1680-90.

13. McDade TW, Ryan CP, Jones MJ, Hoke MK, Borja J, Miller GE, et al. Genome-wide analysis of DNA methylation in relation to socioeconomic status during development and early adulthood. Am J Phys Anthropol. 2019;169(1):3-11.

14. Suderman M, Pappas JJ, Borghol N, Buxton JL, McArdle WL, Ring SM, et al. Lymphoblastoid cell lines reveal associations of adult DNA methylation with childhood and current adversity that are distinct from whole blood associations. Int J Epidemiol. 2015;44(4):1331-40.

15. van Dongen J, Bonder MJ, Dekkers KF, Nivard MG, van Iterson M, Willemsen G, et al. DNA methylation signatures of educational attainment. NPJ science of learning. 2018;3:7.

16. Alfano R, Guida F, Galobardes B, Chadeau-Hyam M, Delpierre C, Ghantous A, et al. Socioeconomic position during pregnancy and DNA methylation signatures at three stages across early life: epigenome-wide association studies in the ALSPAC birth cohort. Int J Epidemiol. 2019;48(1):30-44.

17. Benjamini Y, Hochberg Y. Controlling the False Discovery Rate: A Practical and Powerful Approach to Multiple Testing. Journal of the Royal Statistical Society Series B (Methodological). 1995;57(1):289 - 300.

**Additional file 1: Figures**

| **Additional file 1: Figure S1** Trends in publications over time |
| --- |
| **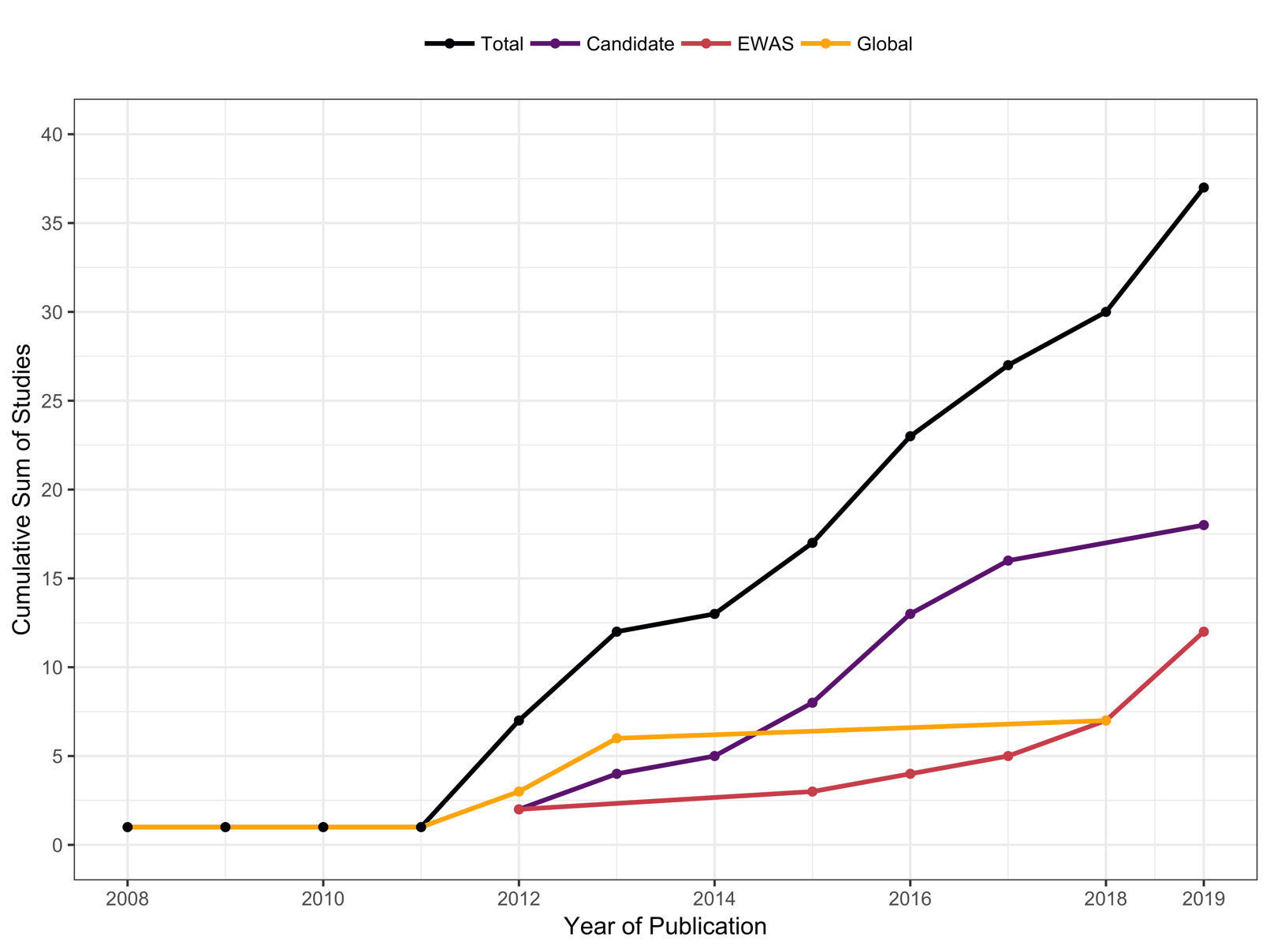** |
| This figure presents the cumulative number of 37 total published studies by year and approach to analysing DNA methylation (DNAm). We conducted an exhaustive literature search from inception until 09/18/2019 using electronic databases PubMed and PsycINFO. This graph illustrates the 37 papers included in the review, beginning with articles published on or after 01/01/2008, the date of the first included published paper identified by our search.  Total, total number of publications for all types of DNAm approaches; Candidate, candidate gene association studies; EWAS, epigenome-wide association studies; Global, global DNAm studies. |

| **Additional file 1: Figure S2** Trends in socioeconomic position (SEP) indicators |
| --- |
| 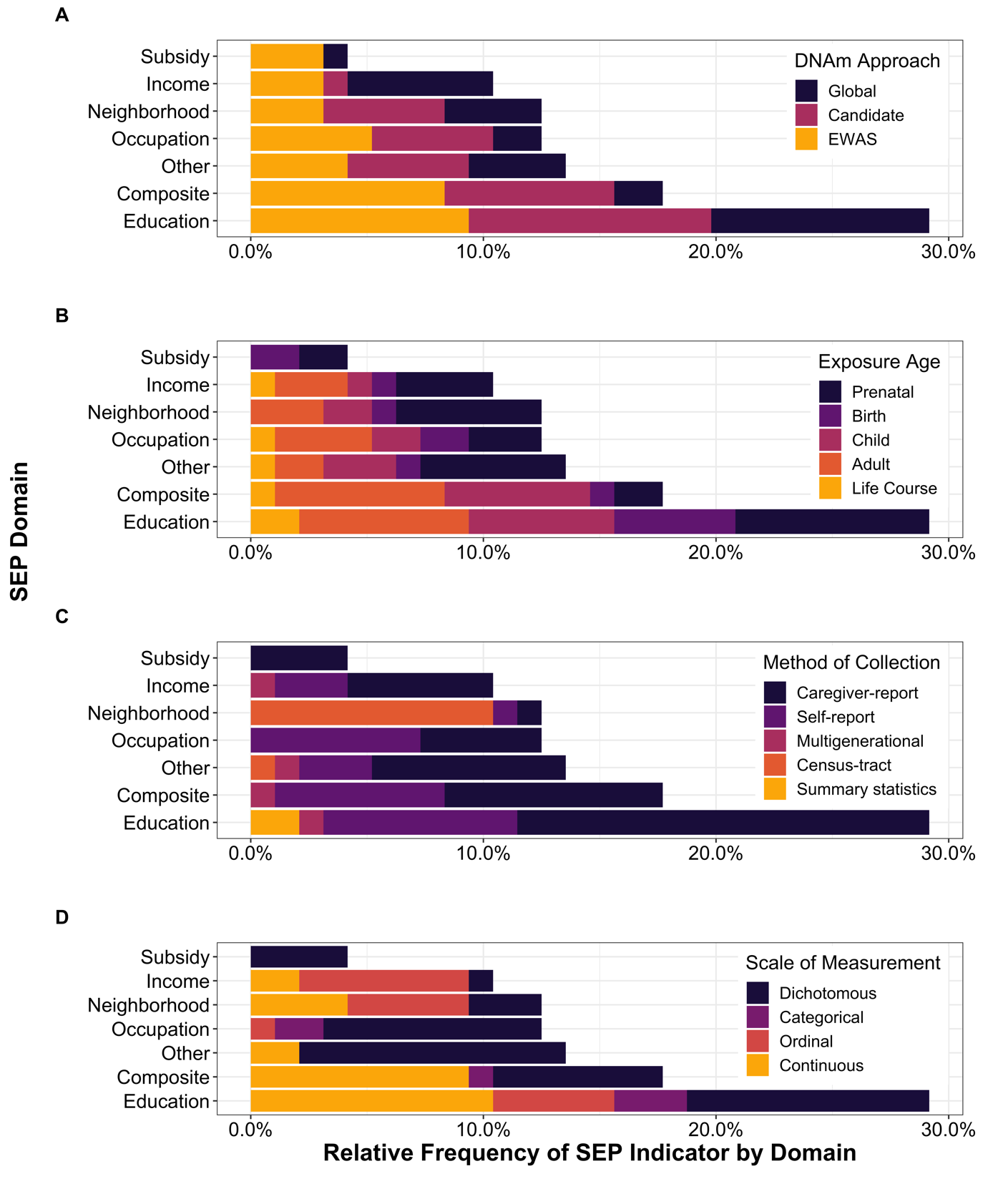 |
| The total number of SEP indicators included in review, categorized by SEP domain and presented by: (A) DNA methylation (DNAm) approach, (B) exposure age, (C) method of collection, and (D) scale of measurement. Relative frequencies are reported, which refer to the percent (%) of SEP indicators by domain over the total number of indicators (n=96) included in the 37 studies examined. For example, measures defined as occupational prestige, employment status, or job title were classified under the *occupation* domain. *Composite* domain included cumulative measure of aggregated SEP measures from multiple SEP domains (e.g., a composite score summarizing occupation, income, and education measures). Measures such as household assets, crowded dwelling, and poverty status were grouped under the *other* domain. For more details on SEP indicators at the individual study level, see **Additional file 2: Tables S1–S3.**  (A) DNAm approach (i.e., approach to analysing DNAm) categorized into three groups: global DNAm, candidate gene, and EWAS (epigenome-wide association study).  (B) SEP exposure age reported by life-course group: prenatal (<0 years), birth (~0 years), child (0-18 years), adult (18+ years), and life course (ages of exposure captured spanned early life to adulthood).  (C) Method of collection used to collect information on SEP indicator categorized by: caregiver report, self-report, multigenerational (both parent and self-reports), census-tract (taken from government official statistical subdivisions of a county of geographical equivalent), and summary statistics.  (D) Scale of measurement used to code SEP indicator for analysis; scales include dichotomous (binary), categorical (unranked), ordinal (ranked), and continuous (numerical). |

| **Additional file 1: Figure S3** Trends in tissue type |
| --- |
| **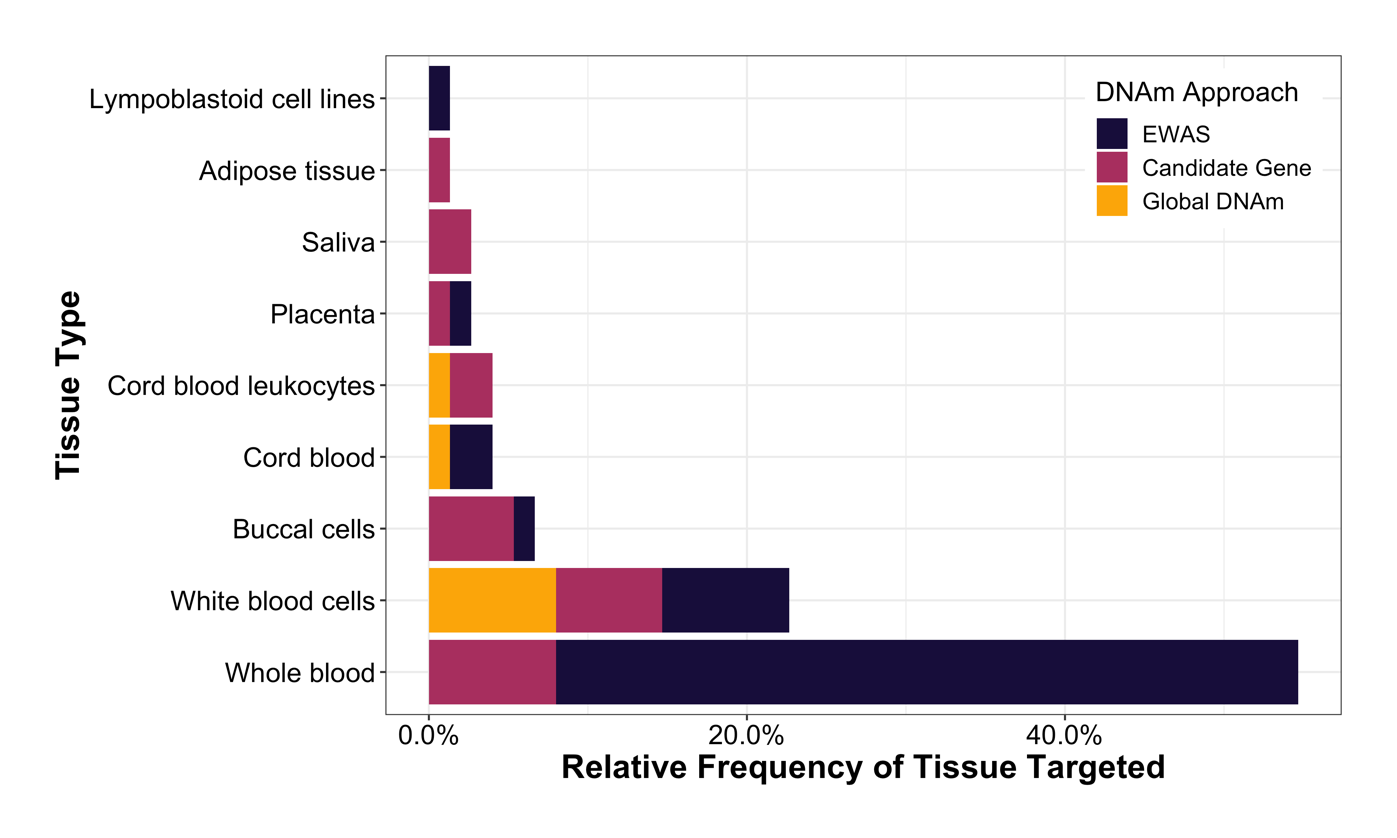** |
| The number of tissue types targeted across 37 studies included in the review, separated by DNA methylation (DNAm) approach (i.e., approach to analysing DNAm): EWAS (epigenome-wide association studies), candidate gene, and global DNAm. Relative frequencies are reported, which refer to the percent (%) out of the 37 studies examined. The most commonly assessed tissue type was whole blood (55%), which was targeted by only EWAS and candidate gene studies, followed by white blood cells (23%), which was evenly targeted between the three DNAm approaches. The remaining tissue types were targeted by less than 1% of studies. Five studies targeted two different tissue types. Tissue types included by each study are presented in **Additional file 2: Tables S1–S3.** |
